# Kidney failure in a child with Pierpont syndrome: a case report highlight challenges in kidney replacement therapy

**DOI:** 10.1186/s12882-026-05152-0

**Published:** 2026-06-27

**Authors:** Sze Wa Wong, Pak Chiu Tong, Josephine Shuk Ching Chong, Lettie Chuk Kwan Leung, Eugene Yu Hin Chan, Alison Lap Tak Ma

**Affiliations:** 1 Paediatric Nephrology Centre, Hong Kong Children’s Hospital, Hong Kong, HKSAR, China; 2 Department of Paediatrics, The Chinese University of Hong Kong, Hong Kong, HKSAR, Hong Kong; 3 Joint Baylor-CUHK Center of Medical Genetics, The Chinese University of Hong Kong, Hong Kong, HKSAR, China; 4 Department of Obstetrics & Gynaecology, The Chinese University of Hong Kong, Hong Kong, HKSAR, China; 5 Department of Paediatrics and Adolescent Medicine, Kwong Wah Hospital, Hong Kong, HKSAR, China

**Keywords:** Case report, Dysplastic kidneys, Kidney failure, Pierpont syndrome, *TBL1XR1*

## Abstract

**Background:**

Pierpont syndrome is a rare autosomal dominant disorder caused by pathogenic variants in *TBL1XR1* gene, characterized by distinctive craniofacial features, plantar lipomatosis, and neurodevelopmental disorders. Urological malformations were reported, but signifcant kidney function impairment has never been described in previous cases.

**Case presentation:**

We report an 8-year-old girl with bilateral dysplastic kidneys identified in neonatal period and progression to kidney failure, requiring peritoneal dialysis from age four. Subsequent genetic evaluation confirmed a diagnosis of Pierpont Syndrome. Her course was complicated by recurrent inguinal hernia, peritoneal dialysate leakage, and respiratory compromise related to central and obstructive apnoea. She has been enlisted for kidney transplantation.

**Conclusion:**

Although causality cannot be established, the concurrence of bilateral dysplastic kidneys and kidney failure in this rare genetic disorder raises important clinical questions. The case also highlights the importance of mutlidisciplinary care in dialysis and transplant planning. Further studies are required to clarify the potential role of *TBL1XR1* gene in kidney development and long-term outcomes in Pierpont syndrome.

## Background

Pierpont syndrome (PS) is a rare genetic disorder first described in 1998 by Dr Pierpont [1]. Two boys from unrelated families who suffered from global developmental delay shared strikingly similar dysmorphic features. These included microcephaly, high forehead, midface hypoplasia, large ears, depressed nasal bridge, and congenital fat pads over palms and heels. Since the first publication, there have been fewer than 20 cases reported worldwide [1–11]. This condition was subsequently linked to pathogenic variants in *TBL1XR1* gene [2]. While most patients suffered from a diverse spectrum of neurological manifestations, some had also been reported with urinary tract anomalies. However, these congenital abnormalities were mild and kidney function was well preserved in all these children [2, 3, 10, 11]. To date, progression to kidney failure has not been described in individuals with Pierpont Syndrome.

## Case presentation

Our patient is an eight-year-old girl, the only child of a non-consanguineous Chinese couple. Both parents were healthy. There was no family history of congenital anomalies of the kidney and urinary tract (CAKUT) or kidney failure. Pregnancy was not complicated by maternal diabetes, hypertension, autoimmune disease, infection, or known teratogenic medication exposure. Antenatal ultrasonography demonstrated oligohydramnios and intrauterine growth restriction in the second trimester. No other structural abnormalities were documented in the available antenatal records. She was delivered at 35 weeks of gestation by elective caesarean section in the setting of fetal growth restriction. Her birth weight, head circumference, and body length were 2.13 kg (10th centile), 32 cm (25th–50th centile), and 41 cm (< 3rd centile), respectively. Central hypotonia and non-specific dysmorphic features were noted at birth. These included prominent forehead, macrotia, bilateral up-slanting and deep-seated eyes, anteverted nares and thick fingers and toes. Despite the presence of oligohydramnios, no clinical features of Potter sequence were identified. She required a short period of invasive ventilatory support for perinatal pneumonia.

Abnormal kidney function was first recognized incidentally on day 5 of life, with a peak serum creatinine of 370 µmol/L (reference: 44–80 µmol/L) and urea up to 27 mmol/L (reference: 1.4–6.8 mmol/L), while electrolytes remained normal. Ultrasound of the urinary system on day 6 revealed bilaterally small kidneys measuring 2 cm, with loss of corticomedullary differentiation. Urine output was preserved at 2–4 mL/kg/hour without diuretics, and blood pressure was normal. She was managed conservatively with fluid control and nutritional support resulting in a fall of serum urea to below 10 mmol/L. Renal function improved partially two weeks later, with serum creatinine stabilising at 120–130 µmol/L and urea at 8–12 mmol/L. She was followed closely at a regional paediatric hospital and referred to our centre at one year of age, when her estimated creatinine clearance had declined to 14 mL/min/1.73 m².

**Fig. 1:**
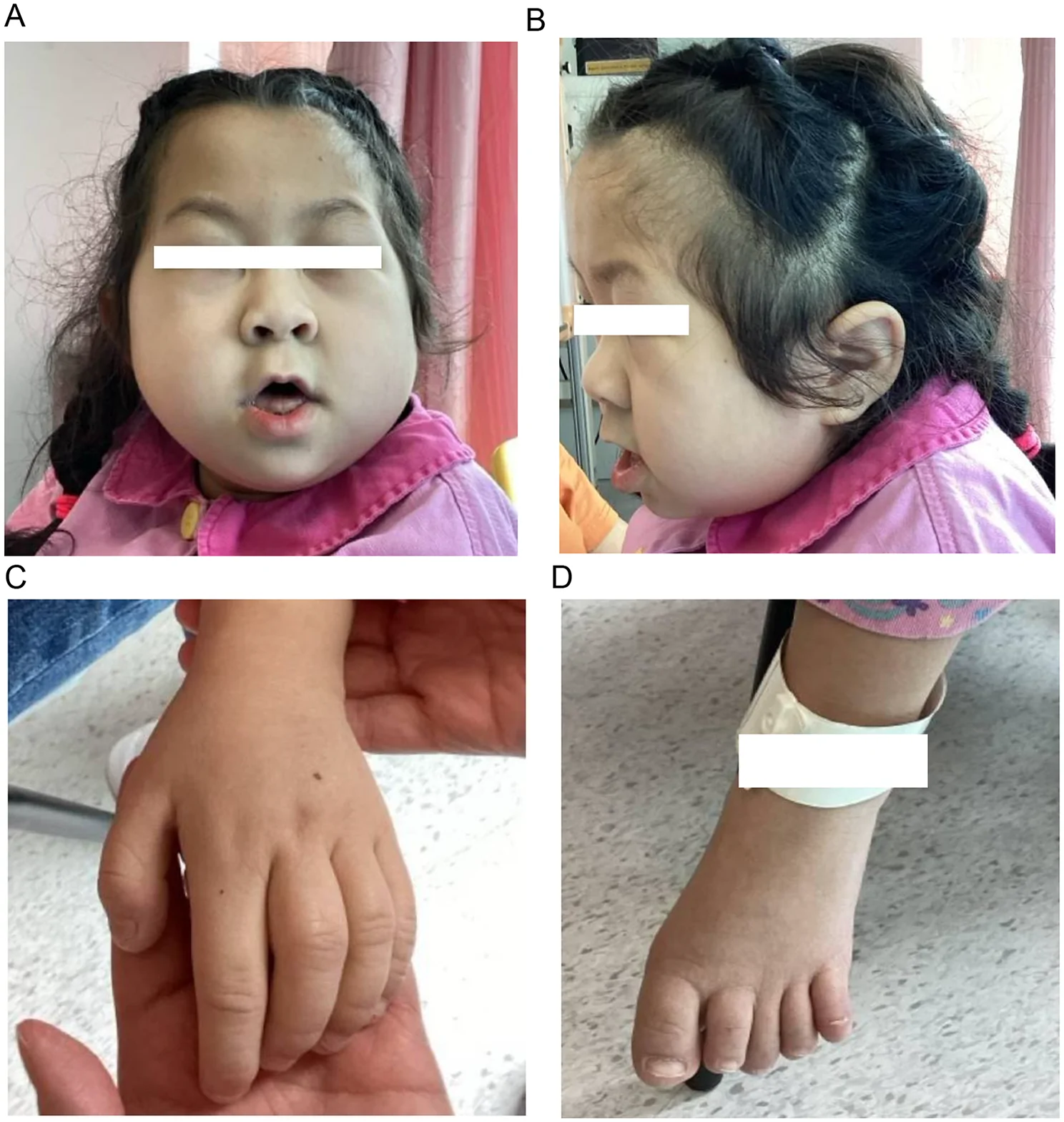
Facial and extremity features of the patient. **A** & **B**: Facial features include short palpebral fissure, deep-set eyes, broad nasal ridge, thin vermillion, prominent ears. **C** & **D**: short and broad hands & feet

Neurodevelopmental concerns were evident from four months of age, when she was noted to have global developmental delay. She also developed moderate to severe sensorineural hearing loss and required gastrostomy feeding for oropharyngeal dysphagia and faltering growth. At five years of age, her developmental age was assessed as equivalent to a one-year-old, with subsequent stagnation. Additional comorbidities included obstructive sleep apnoea (OSA) and central apnoea, which were managed with tonsillectomy and nocturnal non-invasive positive pressure ventilation. Her dysmorphic features became more pronounced over time, including deep-set eyes, short palpebral fissures, a broad nasal bridge, and short, broad hands and feet (Fig. 1). Pierpont syndrome was suspected, and whole-exome sequencing (WES) identified a heterozygous de novo pathogenic missense variant *c.1337 A > G (p. Tyr446Cys)* in the *TBL1XR1* gene. Both parents were tested negative for this variant. No additional pathogenic or likely pathogenic variants associated with CAKUT were identified.

**Fig. 2:**
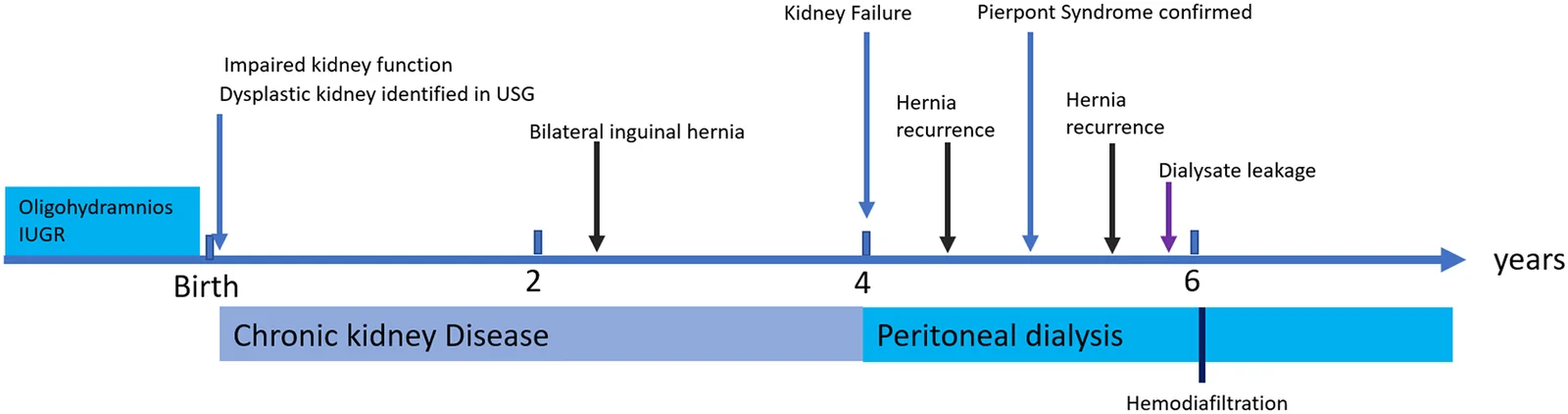
Timeline of clinical course

She progressed to kidney failure by the age of four and commenced peritoneal dialysis (PD). She had previously undergone hernioplasty for bilateral indirect inguinal hernia (IIH). Both deep inguinal rings were confirmed closed at the time of Tenckhoff catheter insertion. Peritoneal dialysis was initiated four weeks postoperatively with gradual escalation of fill volume. Despite this, she developed recurrent bilateral IIH. Dialysis was further complicated by dialysate leakage from the exit site at a relatively low fill volume of 550 mL (800 mL/m²). She was temporarily switched to hemodiafiltration (HDF) during reinsertion of the Tenckhoff catheter and a second hernioplasty. However, HDF was technically challenging. Because of her small body size, vascular calibre was limited, making it difficult to maintain an adequate extracorporeal blood flow. In addition, severe intellectual disability limited her ability to cooperate during HDF sessions, resulting in frequent interruption of dialysis and requirement for sedation. Consequently, PD was resumed using a prolonged regimen of 10 cycles over 12 h with a restricted fill volume of 500 mL (700 mL/m²). Dialysis adequacy was achieved, with Kt/V of 1.89. She remains normotensive and euvolemic and is currently listed for kidney transplantation (Fig. 2).

This case report was prepared in accordance with the CARE reporting guideline.

## Discussion

Pierpont Syndrome is an autosomal dominant disease characterized by distinctive facial dysmorphism, plantar lipomatosis, and neurodevelopmental disorders [1]. It is caused by pathogenic variants of the *TBL1XR1* gene on chromosome 3q26 [2, 3, 11]. This gene encodes transducin β-like 1 X-linked receptor 1 (*TBL1XR1*), which interacts with nuclear hormone receptors and regulates gene transcription [2, 3]. While *TBL1XR1* mRNA expression was demonstrated in pituitary, hypothalamus, white and brown adipose tissues, which may help explain the neurological and cutaneous phenotype, such mRNA expression was not observed in kidney tissues [2]. Nevertheless, CAKUT have been reported in children with PS. Crossed renal ectopia has been described in one boy with PS, although his *TBL1XR1* variant differed from that of our patient [10]. In contrast, among eight children with the same variant as our patient, only one developed mild hydronephrosis, and kidney function was preserved in all cases [2, 3].

To our knowledge, this is the first report describing the coexistence of bilateral dysplastic kidneys with detailed longitudinal follow up through progression to kidney failure and subsequent management in a child with Pierpont syndrome. A possible link between TBL1XR1 mutation and kidney dysplasia lies in the Wnt/β-Catenin Signalling pathway. This gene participates in the Wnt/β-catenin transcriptional complex, facilitating recruitment of β-catenin to target gene promoters. Dysregulation of this pathway has indeed been implicated in nephrogenesis and renal fibrosis through over-stabilization of β-Catenin and down-stream transcription pathway [11, 12]. Although a causal relationship between TBL1XR1 variants and renal dysplasia cannot be established from a single case, reporting such unusual coexistence remains clinically important, particularly in guiding renal surveillance and multidisciplinary management in children with complexed syndromic disorders.

Our case report also illustrates how Pierpont syndrome–related comorbidities can complicate kidney replacement therapy (KRT). Like other neurodevelopmental or syndromic disorders, hypotonia and connective-tissue laxity in Pierpont Syndrome may predispose patients to recurrent hernia and dialysate leakage. Although these complications are not uncommon in young children undergoing PD, the occurrence of dialysate leakage and recurrent hernia despite previous repair and at relatively low FV may suggest increased susceptibility in our patient. Neurodevelopmental impairment and dysphagia further complicated nutritional optimisation and catheter care. In addition, sleep-disordered breathing increased the risk of respiratory compromise during peritoneal dialysis. Furthermore, severe faltering growth and small body size limited vascular calibre and consequently restricted haemodialysis catheter choice and extracorporeal blood flow, often resulting in suboptimal HDF delivery. These issues emphasize the importance of individualized dialysis regimens and anticipating specific complications in children with syndromic conditions.

Kidney transplantation presents additional challenges. Potential risks include impaired wound healing, vascular anastomotic fragility, and uncertainty regarding the long‑term prognosis of PS. The theoretical oncogenic potential of some *TBL1XR1* variants also raises concerns over the safety of lifelong immunosuppression. TBL1XR1 protein has been implicated in multiple signalling pathways affecting tumorigenesis, e.g. the Wnt/β-Catenin Signalling pathway, etc. and somatic *TBL1XR1* mutations have been associated with lymphoma and several solid organ malignancies [11–16]. Since the *TBL1XR1* variant in our patient has been hypothesized to exert a dominant negative effect, it is possible that the inhibitory ability of this protein may be compromised, leading to increased tumorigenic risks, although such a mechanism has not been definitively established [2]. Furthermore, it remains unclear whether germline variants confer similar oncogenic potential to that observed in somatic variants. Nonetheless, tumour development has been reported in PS. A six-month old boy with the same variant as our patient was reported to have atypical choroid plexus papilloma [5]. While published data are extremely limited, thorough multi-disciplinary perioperative planning, vigilant monitoring, and open discussion with families remain essential.

Future directions include clarifying the role of *TBL1XR1* in kidney development. Collaborative reporting of additional cases will be crucial to determine whether kidney diseases represent a genuine component of the Syndrome phenotype or a coincidental finding. Such work may also address prognostic questions, including variant‑specific oncogenicity, which is directly relevant to transplant counselling and long‑term follow‑up.

## Conclusion

Kidney failure can occur in Pierpont Syndrome. Dialysis and subsequent transplantation for this group of patients is challenging because of the related complications and co-morbidities. Early detection and a multi-disciplinary care involving nephrologists are important in optimizing clinical outcome. Further studies and reports are required to better understand the disease mechanism and phenotypic spectrum.

## Data Availability

No datasets were generated or analysed during the current study.

## Abbreviations

CAKUT: Congenital Anomalies of the Kidney and Urinary Tract
HDF: Hemodiafiltration
IIH: Indirect inguinal hernia
KRT: Kidney Replacement Therapy
OSA: Obstructive Sleep Apnoea
PD: Peritoneal Dialysis
PS: Pierpont Syndrome
TBL1XR1: Transducin β-like 1 X-linked receptor 1
WES: Whole exon sequencing
